# Targeting CD44v6 for fluorescence-guided surgery in head and neck squamous cell carcinoma

**DOI:** 10.1038/s41598-018-28059-9

**Published:** 2018-07-11

**Authors:** Julia Odenthal, Mark Rijpkema, Desirée Bos, Esther Wagena, Huib Croes, Reidar Grenman, Otto Boerman, Robert Takes, Peter Friedl

**Affiliations:** 1 Radboud University Medical Center, Department of Otorhinolaryngology and Head and Neck Surgery, Nijmegen, The Netherlands; 2 Radboud Institute for Molecular Life Sciences, Department of Cell Biology, Nijmegen, The Netherlands; 3 Radboud University Medical Center, Department of Radiology and Nuclear Medicine, Nijmegen, The Netherlands; 4 Department of Otorhinolaryngology-Head and Neck Surgery, Turku University and Turku University Hospital, Turku, Finland; 5 UT MD Anderson Cancer Center, Genitourinary Medical Oncology – Research, Houston, TX USA; 6 Cancer Genomics Center, Utrecht, The Netherlands

## Abstract

Head and neck squamous cell carcinoma (HNSCC) is an often highly invasive tumor, infiltrating functionally important tissue areas. Achieving complete tumor resection and preserving functionally relevant tissue structures depends on precise identification of tumor-free resection margins during surgery. Fluorescence-guided surgery (FGS), by intraoperative detection of tumor cells using a fluorescent tracer, may guide surgical excision and identify tumor-positive resection margins. Using a literature survey on potential surface molecules followed by immunohistochemical validation, we identified CD44 variant 6 (CD44v6) as a constitutively expressed antigen in the invasion zone of HNSCC lesions. The monoclonal anti-CD44v6 antibody BIWA was labeled with both a near-infrared fluorescent dye (IRDye800CW) and a radioactive label (Indium-111) and dual-modality imaging was applied in a locally invasive tumor mouse model. BIWA accurately detected human HNSCC xenografts in mice with a tumor uptake of 54 ± 11% ID/g and invasion regions with an accuracy of 94%. When dissected under clinical-like conditions, tumor remnants approximately 0.7 mm in diameter consisting of a few thousand cells were identified by fluorescence imaging, resulting in reliable dissection of invasive microregions. These data indicate that CD44v6 is a suitable target for reliable near-infrared detection and FGS of invasive HNSCC lesions *in vivo*.

## Introduction

Head and neck squamous cell carcinomas (HNSCCs) are invasively growing tumors in a functionally delicate and important area with an overall five-year survival rate below 60%^1^. Surgical treatment of HNSCC aims to completely remove both tumor and marginal invasion zones, and tumor-free resection margins represent a critical prognostic parameter for reducing tumor recurrence and improving overall survival^2^. In functionally critical areas, such as the head and neck region, maximizing surgical margins has to be weighed against loss of functional tissue resulting in compromised quality of life post surgery^3^. Therefore, developments such as intraoperative histology and image-guided surgery aim to more accurately delineate the tumor border during surgery, enabling more accurate tumor resection. As complementary strategy fluorescence-guided surgery (FGS) aims to intraoperatively detect a fluorescent tracer after selective accumulation in tumor tissue and enables preclinical and clinical detection of cancer lesions eventually resulting in improved progression-free survival after surgery^4^. Improved signal detection has been achieved by conjugating fluorophores in the near-infrared (NIR) range of 650–900 nm with targeting antibody binding extracellular epitopes preferentially expressed on tumor cells, allowing macro- and microscopic detection of even small tumors and tumor subregions^5^. This resulted in a range of cancer-targeting antibodies developed for FGS surgery to validate molecular targets, establish conjugate safety and develop sensitive imaging devices^6–8^. In clinical trials safe tracer administration and subsequent identification of cancers in the sub-millimeter resolution have been demonstrated^4,9–11^. Potential shortcomings of FGS, however, include inhomogeneous antigen expression and/or tissue distribution of the antibody as well as non-specific antibody uptake in peritumor tissue and limited tumor detection due to high background fluorescence^4,12^.

First-generation FGS of HNSCC focused on the epidermal growth factor receptor (EGFR), based on its prominent expression in HNSCC and efficient *in vivo* detection of tumors and metastasis in preclinical studies^13–15^. A fluorescently-labeled anti-EGFR antibody (cetuximab) is clinically well tolerated and efficiently differentiates tumor from normal tissue^9,10^. However, reliable cetuximab-based FGS is hampered by uncertain sensitivity and specificity, as a conseqeunce of variable antigen expression in tumors and high binding of cetuximab to normal tissues (tumor stroma, liver, skin, a.o.)^16^. Thus, identifying antigens with a more tumor restricted expression remains pertinent to reliably and selectively visualize HNSCC tumor regions.

To reliably detect the invasion zone of HNSCC, we performed a literature survey and tested the presence of a range of potential antigens (over-)expressed in HNSCC including c-Met, CD44 variant 6 (CD44v6), E-cadherin, epidermal growth factor receptor (EGFR), extracellular matrix metalloproteinase inducer (EMMPRIN/CD147) and epithelial cell adhesion molecule (EpCAM). We identify CD44v6 as candidate and apply anti-CD44v6 antibody BIWA for sensitive detection of the invasion margins in HNSCC in a preclinical mouse model.

## Results

### CD44v6 expression in invasive HNSCC

**Figure 1:**
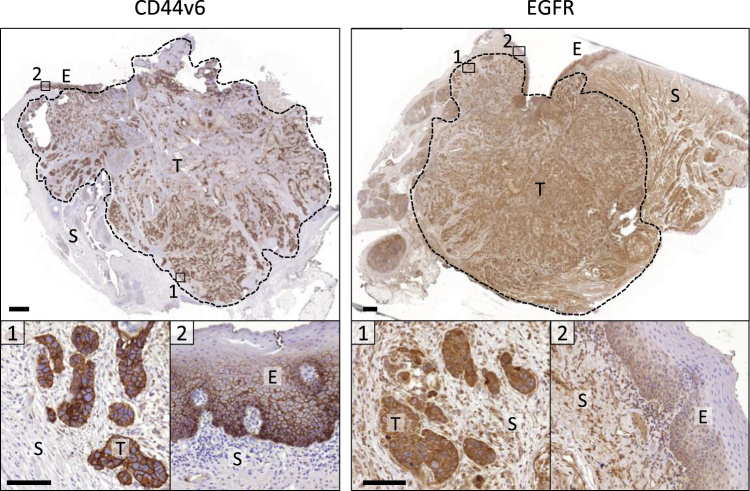
Expression of CD44v6 and EGFR in primary human HNSCC samples. Tumor (T), normal epithelium (E), stroma (S). Dotted lines mark the tumor edge. Representative samples from 7 (CD44v6) or 5 (EGFR) independent tumors. Scale bars indicate 1000 µm (overview) and 100 µm (zoom).

To identify surface markers in HNSCC patient material which reliably detect the margin of invasion and, hence, might be suitable for FGS, we applied comparative immunohistochemistry on human tumor samples. Candidate cell surface proteins, including c-Met, CD44v6, E-cadherin, EGFR, EMMPRIN and EpCAM, were identified based by a literature survey focusing on the percentage of positive tumors, the homogeneity of expression within the same tumor and whether the protein was expressed on the epithelium or the tumor stroma (Suppl. Table 1). As further criteria for marker selection, extracellular cell-surface localization and expression level and variability in HNSCC were considered. Additionally, the availability of a monoclonal antibody with established low toxicity profile and imaging application in clinical trials was taken into consideration. Approximately 97% of HNSCCs were positive for CD44v6 followed by EGFR (85%) and lower frequencies for the other markers. CD44v6 was consistently present throughout the tumor with defined membrane staining, but reduced expression in keratinized or necrotizing areas in the tumor core (Fig. 1). EGFR and c-Met showed a strong expression throughout the tumor similar to CD44v6 (Fig. 1; Suppl. Fig. 1). Likewise, EMMRPIN showed reliable expression throughout the lesion albeit with lower intensity (Suppl. Fig. 1), whereas E-cadherin and EpCAM expression were less reliable with notable inter-individual variability (Suppl. Fig. 1). Whereas for CD44v6 and EMMPRIN the signal was near-exclusively tumor cell specific with only weak background staining from the desmoplastic stroma and adjacent epithelial structures, particularly epidermis and hair follicles, E-cadherin positivity resulted from both tumor-derived and non-transformed epithelial structures (Fig. 1; Suppl. Fig. 1). EpCAM, c-Met and EGFR were also expressed by stromal cells resulting in a high peri-tumor background signal (Fig. 1; Suppl. Fig. 1). The reliable immunohistochemical staining together with published evidence indicated CD44v6 as epitope with abundant expression throughout HNSCC lesions including the invasion zone. For application in FGS, CD44v6-targeting antibodies were previously shown to macroscopically identify CD44v6 expressing epithelial xenograft tumors in mice, including HNSCC (Suppl. Table 1)^17–19^, whereas its suitability for identifying the tumor margin and disseminated invasion zones remain untested. We therefore selected anti-CD44v6 antibody BIWA, the humanized form of which demonstrated safe administration in clinical trials and reliably visualized HNSCC lesions by nuclear imaging^20,21^.

### Expression of CD44v6 in HNSCC cell lines and invasive xenograft tumors in mice

**Figure 2:**
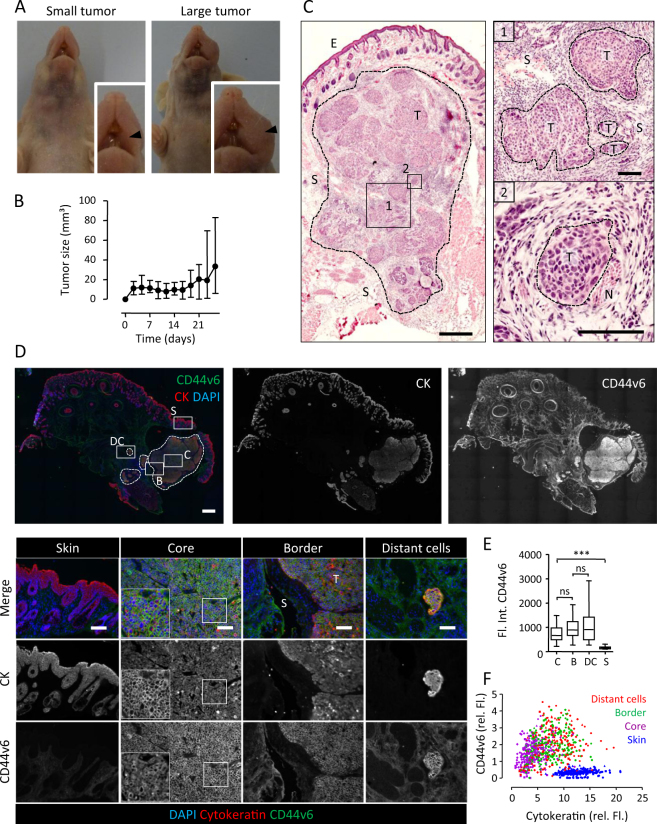
Invasive HNSCC mouse xenograft model for *in vivo* detection of CD44v6. (**A)** Macroscopic detection of a representative small and large tumor in the left cheek of two different mice. Arrowhead indicates the tumor location. (**B**) Growth curve of UT-SCC58 tumors. Data show the means of 12 mice. (**C**) Central section of an UT-SCC58 tumor, showing multifocal invasive tumor islands in the interstitium (1) and in perineural location (2). Scale bars indicate 500 µm (overviews) and 100 µm (zooms). (**D**) Expression of CD44v6 and pan-cytokeratin (CK) in UT-SCC58 tumors. Dotted lines mark the tumor border and tumor islands. Scale bars indicate 500 µm (overviews) and 100 µm (zooms). (**E**) Whole-region analysis of (D). Staining intensity of CD44v6 in different areas analyzed at day 28 after implantation. Data show medians from 12–38 analyzed images per tumor from 4 independent tumors. (**F**) Single cell analysis of (D). Fluorescence intensities  after background substraction for CD44v6 and CK of the same tumors as in (E). 50 cells were analyzed per subregion and geometric symbols represent 5 different tumors. Core of the tumor (C), border of the tumor (B), distant cells (DC), skin (S).

To establish an invasive HNSCC mouse model for FGS, a range of HNSCC cell lines were analyzed for expression of CD44v6 and growth pattern *in vivo*. Consistent with reliable CD44v6 expression in human samples, all HNSCC cell lines expressed CD44v6 at high and homogeneous levels in contrast to two melanoma cell lines serving as negative controls based on previous findings (Suppl. Fig. 2A)^22^. UT-SCC58 showed a low-to-medium level of cell surface expression (50-fold CD44v6/IgG1 signal-to-background ratio; total range between cell lines 30 to 181), which we considered as a representative cell model reflecting non-overstated CD44v6 availability for realistic *in vivo* antibody targeting. As xenograft lesions in mice, UT-SCC58 cells grew within 3–4 weeks to macroscopically visible tumors (Fig. 2A,B) and showed invasive growth pattern characteristics of differentiated HNSCC, including nest-like dissemination in the interstitial tissue (Fig. 2C1) and invasion along nerve fibers (Fig. 2C2). Co-staining of CD44v6 and pan-cytokeratin as epithelial reference marker indicated a relatively uniform expression of CD44v6 throughout the lesion (Fig. 2D). Sub-region analysis of the tumor core, border and invasive cells as well as scoring of CD44v6 intensity at single cell level revealed constant expression in all tumor areas without significant variation (Fig. 2E,F; Suppl. Fig. 2B). Thus, UT-SCC58 xenografts recapitulate the invasive growth and CD44v6 expression patterns of patient samples and represent a suitable preclinical model for FGS of invasive and moderately CD44v6-positive HNSCC tumors.

### BIWA detects CD44v6-expressing tumors *in vivo*

**Figure 3:**
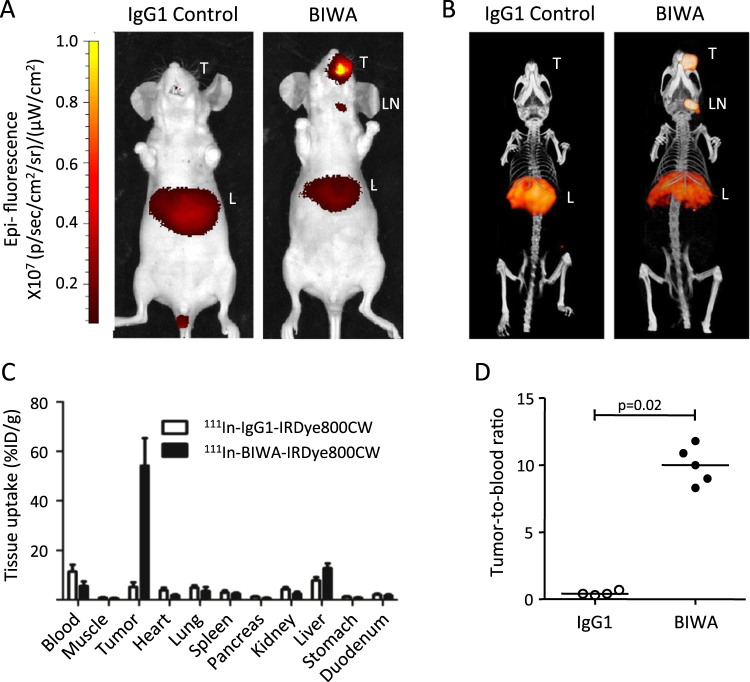
Biodistribution of ^111^In-DTPA-BIWA-IRDye800CW and ^111^In-DTPA-IgG1-IRDye800CW control antibody in mice carrying HNSCC tumors in the left cheek for 28 days and 72 h after i.v. administration. (**A**,**B**) Near infrared fluorescent (NIRF) whole body images of mice n = 9 (A) and corresponding micro SPECT/CT images n = 4 (B). Tumor (T), lymph node (LN), liver (L). (**C**) Biodistribution data shown as percentage injected dose per gram of tissue of both dual-labeled antibodies. (**D**) Tumor-to-blood ratios obtained by ratios from (C).

To detect small metastases and assess the biodistribution of fluorescent tracers, a cohort of UT-SCC58 tumor-bearing mice with a lesion volume of 10–42 mm^3^ received ^111^In-DTPA-BIWA-IRDye800CW or ^111^In-DTPA-IgG1-IRDye800CW isotype-matched control antibody intravenously. Antibody uptake by UT-SCC58 tumors using NIR whole-body fluorescence detection reached a maximum three days after injection (data not shown), similar to the bioavailability kinetics reported for other antibodies and HNSCC tumor models^19,23^. ^111^In-DTPA-BIWA-IRDye800CW showed a most prominent signal originating from the tumor and weaker signal originating from the left cervical lymph node and the liver (Fig. 3A). In comparison, fluorescent signals from ^111^In -DTPA-IgG1-IRDye800CW were detected in the liver and weakly in the tumor (Fig. 3A). SPECT/CT images confirmed this biodistribution profile, whereby one mouse also showed weak signal enrichment in the left cervical lymph node (Fig. 3B). Biodistribution analysis revealed a mean tumor uptake of 54 ± 11% ID/g for ^111^In-DTPA-BIWA-IRDye800CW compared to 5 ± 2% ID/g for IgG1 control antibody conjugate (Fig. 3C). Uptake of dual-labeled BIWA in other organs was considerably lower, quantified as total amount (Fig. 3C) and tumor-to-blood ratio (Fig. 3D). Despite yielding a positive signal (Suppl. Fig. 3A) the cervical lymph nodes lacked pan-cytokeratin positive tumor nests (Suppl. Fig. 3B,C) and thus were considered tumor-free. Most likely, false-positive detection in lymph nodes resulted from uptake of BIWA by macrophages via Fc-receptors, as described for other tumor models^24^. Thus, systemically applied dual-labeled BIWA accumulates in CD44v6-expressing tumors with high selectivity.

### BIWA detects invasive tumor regions

**Figure 4:**
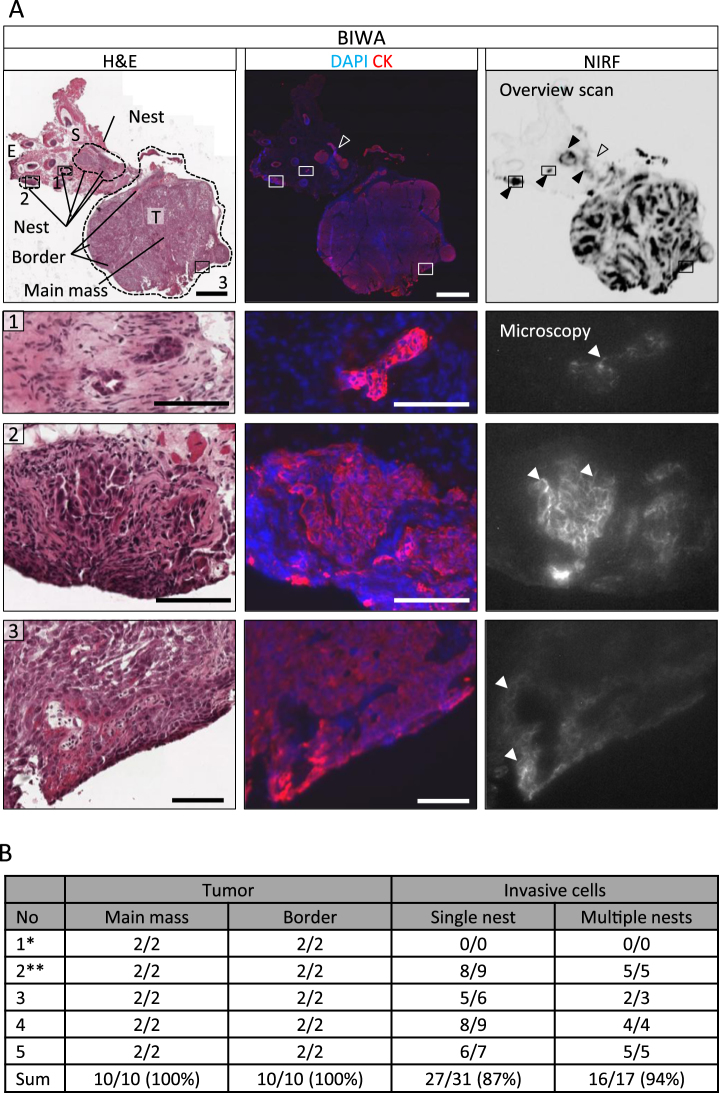
Detection of invasive tumor regions by dual-labeled BIWA 3 days after systemic administration. (**A**) Central, serial sections of a tumor (H&E staining, CK staining and NIRF signal). Images show invasive tumor islands (1, 2, dotted line) adjacent to the main tumor (3, dotted line). Tumor (T), normal epithelium (E), stroma (S). Black arrowheads indicate fluorescent positive islands, the transparent arrowhead a negative/weakly stained island. White arrowheads highlight membranous staining. Scale bars indicate 1 mm (overview) and 100 µm (zoom). (**B)** Scoring of NIRF signal intensity for tumor core, border and invasive cells including isolated nests and clusters of adjacent islands. Data represent scoring of two sections per tumor in 5 mice. ^*^Example shown in Suppl. Fig. 4, ^**^Example shown in Fig. 4A.

To determine whether dual-labeled BIWA is able to detect the invasive tumor margin, we performed sub-region immunohistochemistry and NIR fluorescence scans after systemic administration of ^111^In-DTPA-BIWA-IRDye800CW. Both, the tumor core and invasion zones were macroscopically positive for the near-infrared signal, whereas the surrounding stroma showed a low background signal (Fig. 4A; Suppl. Fig. 4). Microscopic analysis revealed cell surface localization of BIWA label on tumor cells (Fig. 4A, white arrowheads), consistent with antigen-dependent accumulation after *in vivo* administration. Sub-region co-localization analysis of the CD44v6 NIRF signal after *in vivo* administration and pan-cytokeratin immunofluorescence showed that BIWA detected > 85% of the CK-positive tumor cell nests (Fig. 4A, black arrowheads; Fig. 4B). Notably, occasional BIWA-negative tumor clusters were frequently located in direct vicinity (below ~ 250 µm distance) of BIWA-positive regions (Fig. 4A, open arrowheads) and thus would co-localize when viewed by intraoperative microscopic surgery with a sub-millimeter resolution. Including such co-localized events, 94% of the invasion regions were detected by ^111^In-DTPA-BIWA–IRDye800CW (Fig. 4B). No signal from IgG1 control antibody was detected in both tumor cells or the tumor stroma by histological scoring (Suppl. Fig. 4). Taken together, systemically applied dual-labeled BIWA detects all tumor regions and > 90% of invasive zones.

### Preclinical FGS: sensitive detection of tumor cells

**Figure 5:**
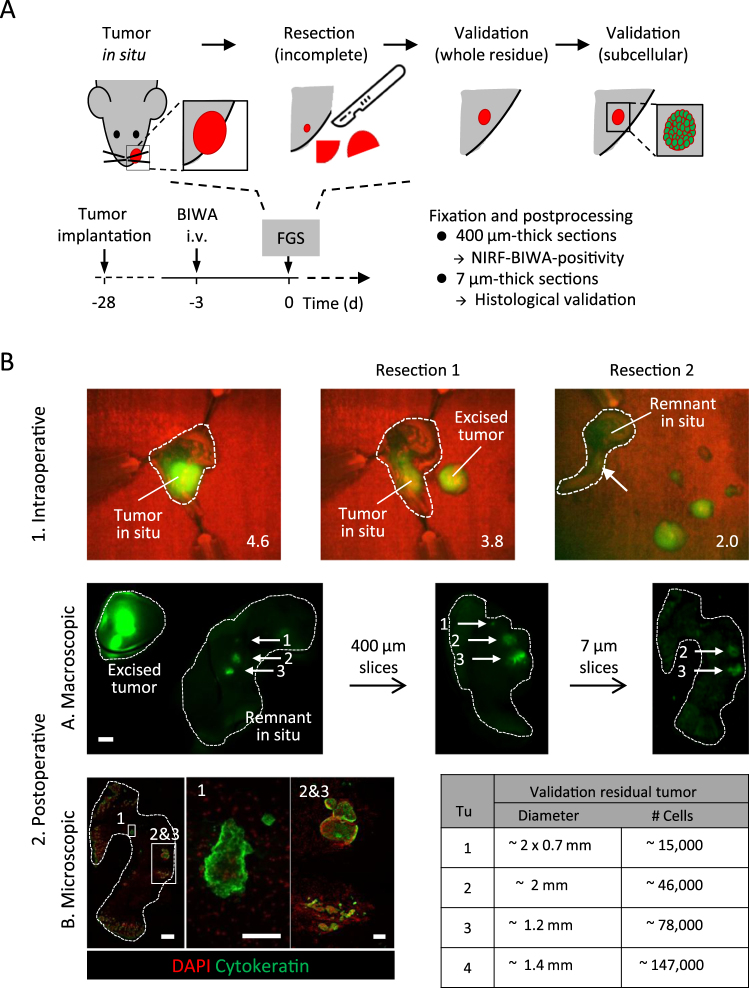
Preclinical FGS using BIWA-IRDye800CW and post-resection validation. (**A**) Experimental procedure of step-wise tumor resection, *in situ* detection and post-fixation analysis. (**B**) (1) UT-SCC58 tumors 28 days after implantation into mice were monitored by intraoperative fluorescence imaging (QMI Spectrum) followed by step-wise FGS. Dotted lines mark the mouse and tumor tissue and the arrow indicates weak fluorescent signal from tumor remnant *in situ*. Numbers show tumor-to-background ratios. (2) Post-operative high-sensitivity NIR fluorescence analysis of excised tissues and *in situ* remnant using the Odyssey CLx flatbed scanner. The scale bar indicates 1 mm. Further processing and re-scanning of the tumor remnant as 400- and then 7-µm thick sections. Immunofluorescence of a NIRF-positive 7 µm section of the remnant. Scale bars indicate 500 µm (overview) and 100 µm (zoom). The total estimated remnant tumor cells and size are shown for four experiments.

FGS experiments were performed using systemically applied BIWA-IRDye800CW detected by the intraoperative QMI Spectrum fluorescence imaging system (Quest Medical Imaging), to approximate signal intensity and detection of the tumor margin under clinical-like conditions. To simulate incomplete tumor resection and estimate the size of minimal remnants of the resection margin based on the BIWA-IRDye800CW label, step-wise FGS was performed until a minimal-residual fluorescent signal was left *in situ* (Fig. 5A). Subsequently, remnants were detected by high-sensitivity NIRF imaging followed by high-resolution immunohistochemistry to validate the presence and amount of tumor cells (Fig. 5A). Post-operative immunohistochemistry showed that NIR fluorescence-positive regions also stained positively for pan-cytokeratin (Fig. 5B), confirming high selectivity of CD44v6 for the detection of tumor cells. The estimated residual tumor masses, which could still be detected by BIWA-IRDye800CW in minimal lesions, were 0.7–2 mm in size and contained few thousand to hundred thousand cells, based on NIR scans and immunohistochemistry of the residual tissue.

## Discussion

We here show that systemically applied BIWA-IRDye800CW provides sensitive detection of HNSCC lesions under clinical fluorescence imaging conditions and allows a precise step-wise removal of the tumor margin with a detection limit in the submillimeter range. CD44v6 is expressed in HNSCC across all tumor stages, with varying trends in T stage and/or nodal status^25–28^. The robust expression of CD44v6 across independent HNSCC lesions and most, if not all, HNSCC tumor cells and the availability of clinically applicable anti-CD44v6 antibody Bivatuzumab emphasize CD44v6 as promising antigen for reliable FGS in HNSCC cancer, enabling visualization of tumor margins and minimal residual lesions. Besides in primary tumors, CD44v6 is expressed in recurrent tumors and lymph node metastases^27,29,30^, therefore administration of ^111^In or IRDye800CW labeled Bivatuzumab should enhance detection of primary as well as secondary and metastatic HNSCC disease. When directly compared, CD44v6 expression is approximately 8-fold higher in HNSCC samples compared to EGFR^31^. CD44v6 is expressed only in a subset of adjacent normal epithelia and absent or only weakly expressed in other, non-tumor tissues^30^, which minimizes background signal, systemic uptake and potential off-target effects. In addition, radiolabeled Bivatuzumab, which targets the same epitope as BIWA, was successfully applied for scintigraphic *in vivo* imaging of HNSCC tumors in mouse models and patients with low toxicity and immunogenicity^19,32,33^. Tumor uptake of the dual-labeled BIWA in UT-SCC58 tumors with moderate CD44v6 expression was comparable to that of radiolabeled anti-CD44v6 antibodies, while tumor-to-blood ratios even exceeded previously reported results^17,19^.

Using detailed immunohistochemical analysis of CD44v6 in patient material and by *in vivo* targeting of HNSCC xenografts by BIWA, we demonstrate that CD44v6 is strongly expressed in the invasion margin of tumor lesions and thus suitable for precise microscopic FGS mapping of invasive HNSCC. Using minimal lesion analysis, we identify high detection sensitivity of BIWA-IRDye800CW using a clinical fluorescence imaging system detecting as little as sub-millimeter sized residues containing only a few thousand cells. The staining pattern was predominantly cell-surface associated with heterogeneous targeting in the main tumor mass, potentially caused by heterogeneous antibody distribution^34^. However, the consistent labelling of tumor borders and invasive cells indicated that Bivatuzumab may enable reliable intraoperative fluorescent imaging of both, invasion zone and small tumor remnants.

Besides HNSCC, CD44v6 is expressed in most carcinoma types, including lung, skin, cervix, breast and colon^30^. Thus, beyond HNSCC, CD44v6 may serve for probing resection margins in a range of other invasive tumor types. CD44v6 targeting antibodies have achieved reliable NIR fluorescence enrichment *in vivo* in breast cancer xenografts^18^ and immuno-PET imaging in a thyroid carcinoma model^35^. Likewise, ^186^Re-labeled Bivatuzumab was safely administered to patients with early-stage breast cancer, but failed to reliably detect small lesions with low CD44v6 expression^36^. Thus, further studies will be required to establish the application in small lesions with clinically variable CD44v6 expression.

Besides application as a single agent, CD44v6 detection may be combined with EGFR detection, the expression of which, in contrast to CD44v6, increases in poorly differentiated carcinomas^16,30^. Thus, combining CD44v6 and EGFR targeting may increase overall sensitivity for the detection of poorly differentiated carcinomas. Additional epitopes for dual- or multi-targeted FGS may include c-Met and EMMPRIN, based on high expression in HNSCC samples and their presence in the invasive front (Suppl. Table 1). In conclusion, based on its beneficial signal-to-noise ratio and low side effects^32^ Bivatuzumab, as either NIR-fluorescence- or dual-labeled conjugate, shows potential to improve the outcomes of surgical treatment in HNSCC.

## Material and Methods

### Literature survey

A systematic PubMed search was performed to identify potential cell surface markers expressed in the invasion region of HNSCC. The following search strategies were used for a publication period from 1985 to September 2015: protein name + immunohistochemistry + head and neck squamous cell carcinoma; protein name + imaging + head and neck squamous cell carcinoma. As indicator, the percentage of patients positive for the protein of interest was averaged from retrieved articles and, where available, differential expression within different tumor regions was taken into account (tumor core, tumor border, surrounding tumor stroma).

### Immunohistochemistry of patient material

Tissue samples from 7 HNSCC patients were obtained from the Department of Pathology, Radboudumc, Nijmegen. Tumor samples were encrypted and analyzed in an anonymized manner, as approved by the institutional review board and according to national law. Formalin-fixed and paraffin-embedded tissues were cut into sections of 4 µm thickness, mounted onto SuperFrost slides (Thermo Scientific) and dried overnight at 37 °C. Sections were deparaffinized in xylene, rehydrated in graded alcohols to water, quenched of endogenous peroxidases (0.3% H_2_O_2_, 30 min), boiled in citrate buffer for antigen retrieval (pH 6, 13 min), pre-incubated with normal goat serum (20%, 30 min), washed and incubated with primary antibody (4 °C, overnight). The following antibodies were used: monoclonal mouse anti-human E-cadherin (SPM471, 1/300; Thermo Scientific), monoclonal mouse anti-human EpCAM (VU1D9, 1/200; Thermo Scientific), monoclonal rabbit anti-human c-Met (EP1454Y, 1/200; Epitomics), polyclonal rabbit anti-human EGFR (sc-03, 1/200; Santa Cruz); monoclonal mouse anti-human EMMPRIN (sc-21746, 1/200; Santa Cruz) and monoclonal mouse anti-human CD44v6 (VFF-18, 1/1000; AbD Serotec). After washing, sections were incubated with biotinylated secondary goat anti-mouse or anti-rabbit antibody and subsequently with the VECTASTAIN ABC reagent (Vector Laboratories) according to the manufacturer’s protocol. The signal was developed with diaminobenzidine and counterstained with hematoxylin. Sections were dehydrated in ethanol and xylene and mounted with Pertex (Histolab). Negative controls (buffer only) were included in each analysis. Sections were imaged with an automated slide scanner with a 20x, 0.243 µm/pixels objective (Pannoramic 250 Flash II, 3DHITECH).

### Cell culture

HNSCC cell lines from primary human SCCs of cutaneous SCC (UT-SCC12A), tongue (UT-SCC16A and UT-SCC40), glottic larynx (UT-SCC38), supraglottic larynx (UT-SCC42B) as well as from metastases of SCCs of cutaneous SCC (UT-SCC12B), tongue (UT-SCC16B) and transglottic larynx (UT-SCC58) were established at the University of Turku, Finland^37^. Other cells used were FaDu cells (kind gift by the Dept. of Radiation Oncology, University of Technology Dresden) and the human melanoma cell lines A3755M (kind gift from Dr. Menashe Bar-Eli/Dr. Michael Davis, MD Anderson Cancer Center, USA) and MV3^38^. All cells were cultured in Dulbecco’s modified Eagle medium (DMEM, Invitrogen), supplemented with 10% heat-inactivated fetal calf serum (Sigma Aldrich), 100 U/ml penicillin and 100 µg/ml streptomycin (both PAA), 2 mM L-glutamine, 1 mM sodium pyruvate (both Invitrogen/Life technologies) and 20 mM HEPES (Gibco).

### Flow cytometry

2 × 10^5^ HNSCC and melanoma cells were seeded in 6-well plates two days prior to the experiment. Cells were detached with 4 mM ethylenediamine tetraacetic acid (EDTA) and stained on ice with murine anti-human CD44v6 antibody (BIWA), the humanized form of which has been applied clinically (bivatuzumab; BIWA-4)^19,39^ or a human IgG1 istotypic control (Alpha Diagnostic Intl. Inc.) and a secondary goat anti-mouse/human 647 antibody (ThermoFisher Scientific). CD44v6 expression and cell viability co-registered by lack of propidium iodide uptake were obtained by flow cytometry (FACS Caliber) and analyzed using the FCS Express software (version 5).

### Mouse model

The animal experiments were performed in accordance with the guidelines and rules of the Dutch Act on animal experiments (WOD) and was approved by the Animal Welfare body of the Radboud University, Nijmegen (RU-DEC 2014-142). 9 × 10^5^ UT-SCC58 cells suspended in 3 mg/ml Matrigel (total volume 50 µl; BD Biosciences) were injected into the floor of the mouth of BALB/c nu/nu female mice (6–8 weeks old; Charles River Laboratories). Tumors were allowed to grow for 4–6 weeks. Tumor growth over time was measured using a caliper.

### Antibody conjugation

Antibodies were used as either fluorescence-only or dual-labeled conjugates carrying fluorescence and the radioactive isotope ^111^In. Human IgG1 isotypic control antibody (Alpha Diagnostic Intl. Inc.) was dialyzed against phosphate buffered saline (PBS) using the 10,000 Da molecular weight cutoff Slide-A-Lyzer MINI Dialysis device (Thermo Scientific) to remove azide and other additives. To generate NIR fluorescent conjugates, BIWA (1 mg) was incubated with a 3-fold excess of IRDye800CW-N-hydroxysuccinamide (NHS) in 0.1 M sodium carbonate buffer (pH 8.5) for 1 h at room temperature (RT). For fluorescence guided surgery experiments, BIWA was only labeled with IRDye800CW-NHS. The final concentration of the antibody and the molecular substitution ratio were determined spectrophotometrically (Ultrospec 2000 spectrophotometer, Pharmacia Biotech) yielding an antibody:fluorophore ratio of 1:2. BIWA-IRDye800CW was stored in the dark at 4 °C.

For dual-labeling, IRDye800CW-labeled antibodies were conjugated with the chelator *p*-isothiocyanatobenzyl-diethylenetriaminepentaacetic acid (ITC-DTPA) with a 10-fold excess in 0.1 M sodium carbonate buffer (pH 9.5) for 1 h at RT. The solution was dialyzed for 1 week against 0.25 M ammonium acetate (pH 5.5) using a Slide-A-Lyzer cassette with a molecular weight cutoff of 20,000 Da (Thermo Scientific). The final concentration of the antibody and the molecular substitution ratio of the fluorescent dye were determined spectrophotometrically (Ultrospec 2000 spectrophotometer, Pharmacia Biotech) yielding a ratio of 1.4. DTPA-BIWA-IRDye800CW and DTPA-IgG1-IRDye800CW were stored in the dark at 4 °C until further use.

For radiolabeling, DTPA-BIWA-IRDye800CW and DTPA-IgG1-IRDye800CW were incubated with 0.05 MBq of ^111^In (Mallinckrodt) per microgram of antibody in two volumes of 0.5 M 2-(N-morpholino)ethansulfonic acid (MES) buffer (pH 5.5). For SPECT/CT studies antibodies were labeled with 1.5 MBq of ^111^In per microgram of antibody. After incubation of 20 min at RT 50 mM EDTA was added to a final concentration of 5 mM to chelate unincorporated ^111^In. Labeling efficiency was determined by instant thin-layer chromatography on silica gel strips (Agilent Technologies) using 0.15 M citrate buffer, pH 6.0 as the mobile phase. For all preparations, the radiochemical purity of ^111^In-DTPA-BIWA-IRDye800CW and ^111^In-DTPA-IgG1-IRDye800CW exceeded 95%.

Unperturbed immunoreactivity of the antibody conjugates was confirmed as described^40^ with minor modifications. A serial dilution of UT-SCC58 cells was incubated with radiolabeled antibody conjugate (333 Bq equivalent to 6.7 ng antibody per dilution in DMEM medium) for 30 min at 37 °C. To determine nonspecific binding, an excess of unlabeled antibody conjugate was added to a duplicate of the lowest cell concentration. Unbound antibody was removed by washing with DMEM and samples were analyzed in a γ-counter (Wizard; Pharmacia-LKB). For all preparations, the immunoreactive fraction of available antibody exceeded 85%.

### SPECT/CT and fluorescence whole body imaging

Mice bearing UT-SCC58 tumors in the left cheek were injected into the tail vein with either 10 µg of the dual-labeled antibody ^111^In-DTPA-BIWA-IRDye800CW or ^111^In-DTPA-IgG1-IRDye800CW. Three days after injection the distribution of the tracer was determined with a U-SPECT II SPECT/CT scanner (MILabs, Utrecht, The Netherlands) in four mice using a 1.0-mm-diameter multipinhole mouse/rat collimator (2 × 25 min with 44 bed positions). Images were reconstructed by ordered-subset maximization expectation using the MILabs reconstruction software (U-SPECT-Rec, Milabs, Utrecht, The Netherlands) with the following settings: selection of the lower ^111^In photopeak (152–183 keV), corrected for two backgrounds (135–151 keV and 184–211 keV), pixel based OSEM, voxel size 0.4 mm^3^ and 1 iteration over 16 subsets. After SPECT imaging, all mice were euthanized and whole-body fluorescence images were acquired (IVIS Lumina imaging system, Caliper Life Science) using the following settings: F/Stop - 2, excitation filter - 745 nm, emission filter - ICG, field of view - C, 675 nm autofluorescence and background correction, recording time of 1 min and medium binning factor. Images were processed with the IVIS Lumina software.

### Biodistribution analysis

Three days after intravenous administration of the antibodies and whole-body imaging, the biodistribution of ^111^In-DTPA-BIWA-IRDye800CW and ^111^In-DTPA-IgG1-IRDye800CW in body fluids and excised organs of mice were determined. Blood was collected by heart puncture and tissue samples of the tumor lesion, superficial cervical lymph nodes, muscle, heart, lung, spleen, pancreas, kidney, liver, stomach and duodenum were collected, weighed and the radioactivity was measured in a well type γ counter (Wallac 2480 wizard, Perkin Elmer). Radioactivity uptake in each tissue was calculated as the percentage of the injected dose per gram of tissue.

### Immunohistochemistry of mouse tumors and cervical lymph nodes

Frozen tumor sections (7 µm thickness) were fixed in ice-cold methanol (10 min), washed and pre-incubated with normal goat serum (5%, in 1% BSA, 1 h) followed by incubation with primary antibody (overnight, 4 °C). Formalin-fixed cervical lymph nodes and tumors were embedded in paraffin, sectioned (7 µm), mounted onto SuperFrost slides (Thermo Scientific), dried overnight (37 °C), deparaffinized in xylene and rehydrated in graded alcohols to water. Sections were stained with hematoxylin eosin (H&E) or boiled in EDTA/Tris buffer (pH 9, 15 min) for antigen retrieval and pre-incubated with normal goat serum (5%, in 1% BSA, 1 h) followed by incubation with antibody (overnight, 4 °C). The following antibodies were used: polyclonal rabbit anti-human and mouse pan-cytokeratin (CK) (ab9377, 1/100; Abcam); monoclonal murine anti-human CD44v6 (BIWA, 5 µg/ml; kind gift from V. Orian-Rousseau, Karlsruhe Institute of Technology, Germany). After washing, sections were incubated with secondary goat anti-rabbit Alexa Fluor 546 and goat anti-mouse/human Alexa Fluor 647 antibody (Invitrogen) for 2 h at RT and counter stained with DAPI (Sigma). Negative controls were obtained by IgG isotype staining and tumor tissue served as positive controls for lymph node staining. Fluorescence on tissue sections was imaged using a 10x NA 0.25 objective (DMI6000B slide scanner, Leica) and H&E staining using a 20x 0.243 µm/pixels objective (Pannoramic 250 Flash scanner, 3DHITECH).

Digital image analysis was performed using Fiji (software version 1.51k, https://imagej.net/Fiji). Fluorescence intensity of CD44v6 and CK were co-registered for tissue subregions from images containing skin, local invasion, the core or the border of the tumor and each subregion was manually segmented based on topology, DAPI and CK signal. Mean CD44v6 and CK fluorescence intensities in individual cells were obtained using same region of interest, using an area of 51 µm^2^ to sufficiently represent the size of single cells. Fluorescence intensities were normalized by subtracting the mean intensity from five background measurements from tumor-free tissue regions.

### NIR imaging and analysis of mouse tumors and lymph nodes

Fresh-frozen tumor tissue from mice after i.v. administration of ^111^In-DTPA-BIWA-IRDye800CW or ^111^In-DTPA-IgG1-IRDye800CW were sectioned (7 µm thickness) and two positions with at least 400 µm distance were scanned for NIR fluorescence intensity (settings: slide, intensity 2, resolution 21 µm, highest quality; Odyssey CLx, LI-COR Biosciences). Regions of interest identified by fluorescence detection from the Odyssey scans were re-sampled with high resolution by NIR imaging (20x NA 0.8; mono setting, 5 sec acquisition time; Nuance-XR, PerkinElmer). Further subsampling and validation of fluorescence-positive regions obtained by Odyssey scanning were obtained by H&E and CK staining.

### Fluorescence-guided surgery

UT-SCC58 tumor-bearing mice after 28 days of growth in the left cheek received 10 µg of BIWA-IRDye800CW by tail vein injection. Three days later whole-body fluorescence was detected under isoflurane anesthesia followed by euthanasia. For intraoperative fluorescence detection, the tumor-containing cheek including surrounding fluorescence-negative tissue was surgically isolated and monitored in real-time using the Spectrum camera system (Quest medical imaging, Wieringerwerf) under clinically used imaging conditions. To match step-wise tumor resection conditions, sequential removal of the lesion guided by fluorescence was performed, until a weak but nonetheless positive signal *in situ* was obtained, reflecting a minimal tumor residue. Tumor-to-background signals were analyzed using Fiji (software version 1.51k, https://imagej.net/Fiji). For verification of fluorescence, all freshly excised tissue samples including the residual lesion in the cheek *in situ* were subjected to high-sensitivity NIR scanning (instrument settings as described above; Odyssey CLx, LI-COR Biosciences) as native whole-mounts, followed by fixation (10% formalin, over night, RT, in the dark). Images were used to estimate the remnant tumor size. To detect both antibody distribution and topology of the resected lesions, the cheeks were sliced into 400 µm thick sections (Vibratome VT 1000 S, Leica), scanned again for fluorescence (Odyssey CLx), embedded in paraffin, sliced into 7 µm thick sections and scanned for NIR fluorescence (Odyssey). Subsequently, samples were stained for CK and DAPI or H&E and the amount of tumor cells in the remnant was estimated by counting the tumor cells of one central slide multiplied by the volume of cytokeratin positive sections, taking into account that each nucleus is present on every 1.7 section based on a mean diameter of a tumor cell nucleus of 12.3 µm (averaged from 50 UT-SCC58 cell nuclei).

### Statistics

Statistical analysis was performed using the non-parametric Mann-Whitney test with a post-hoc correction for multiple comparisons (p ≤ 0.05/N).

### Data Availability

Datasets generated during and/or analyzed during the current study are available from the corresponding author on reasonable request.

## Electronic supplementary material

Supplementary data
