# Supplementary material for: Targeting CD44v6 for fluorescence-guided surgery in head and neck squamous cell carcinoma
Source: Sci Rep. 2018 Jul 11;8:10467. doi: 10.1038/s41598-018-28059-9 (PMC6041314; doi:10.1038/s41598-018-28059-9)
Supplement: Supplementary file 1 — Supplementary data [file 41598_2018_28059_MOESM1_ESM.pdf]

Supplementary information

Targeting CD44v6 for fluorescence-guided surgery in head and neck squamous cell carcinoma

Julia Odenthal[1,2], Mark Rijpkema[3], Desirée Bos[3], Esther Wagena[2], Huib Croes[2], Reidar Grenman[4], Otto Boerman[3], Robert Takes[1*], Peter Friedl[2,5,6*]

[1] Radboud University Medical Center, Department of Otorhinolaryngology and Head and Neck Surgery, Nijmegen, The Netherlands
[2] Radboud Institute for Molecular Life Sciences, Department of Cell Biology, Nijmegen, The Netherlands
[3] Radboud University Medical Center, Department of Radiology and Nuclear Medicine, Nijmegen, The Netherlands
[4]Department of Otorhinolaryngology-Head and Neck Surgery, Turku University and Turku University Hospital, Turku, Finland
[5] UT MD Anderson Cancer Center, Genitourinary Medical Oncology – Research, Houston, TX, USA
[6] Cancer Genomics Center, The Netherlands

Supplementary Figure 1

A

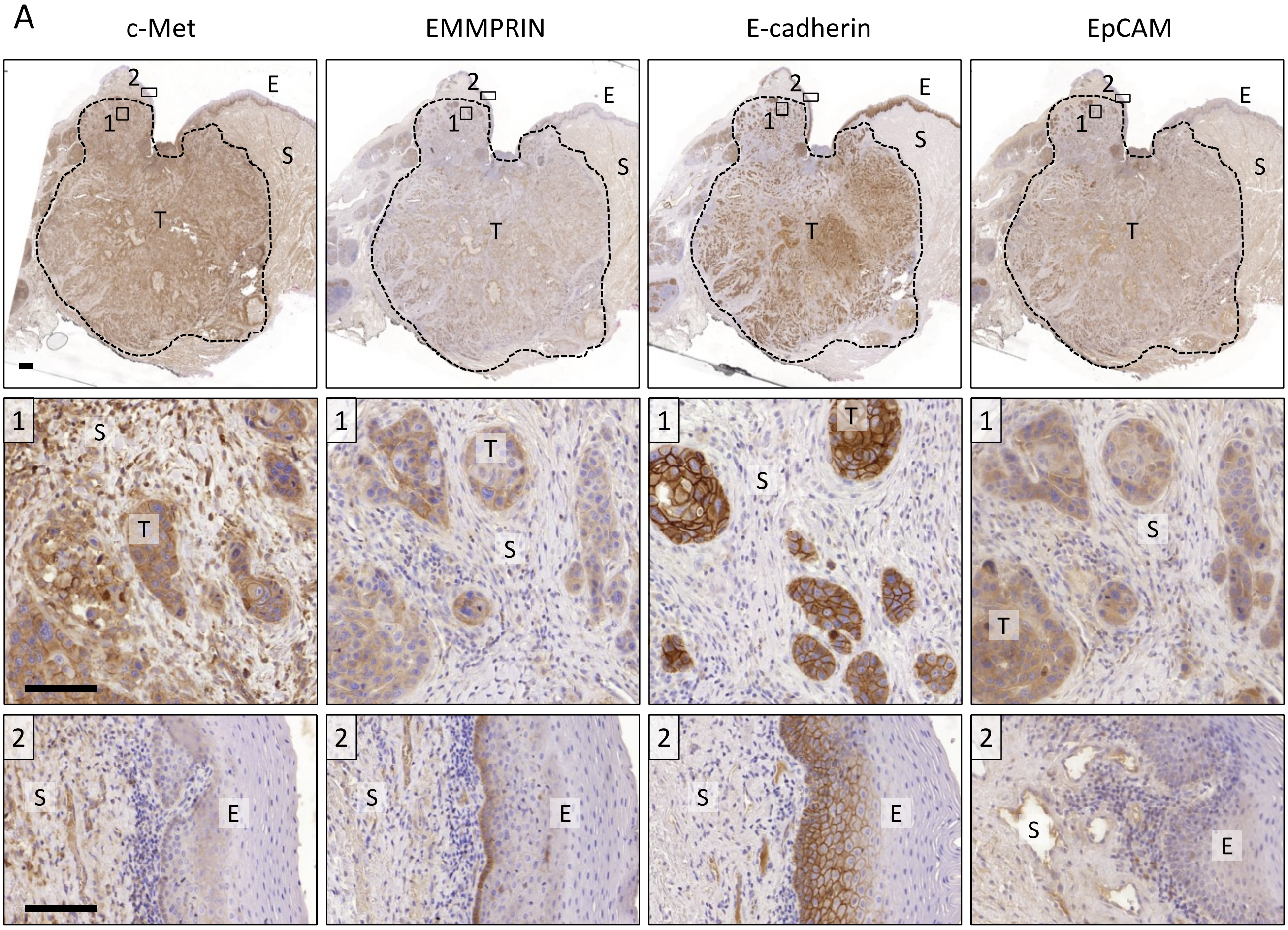

B

	C-Met	CD44v6	E-cadherin	EGFR	EMMPRIN	EpCam
Tumor	Strong (5/5)	Strong (7/7)	Strong (4/5)<br>Weak (1/5)	Strong (5/5)	Strong (3/4)<br>Weak (1/4)	Strong (1/5)<br>Weak (2/5)<br>Neg (2/5)
Tumor border	Positive	Positive	Positive	Positive	Positive	Positive
Epithelial tissue	Positive	Positive	Positive	Positive	Positive	Positive
Background stroma	Strong	Weak	Weak	Strong	Weak	Strong

Supplementary Figure 2

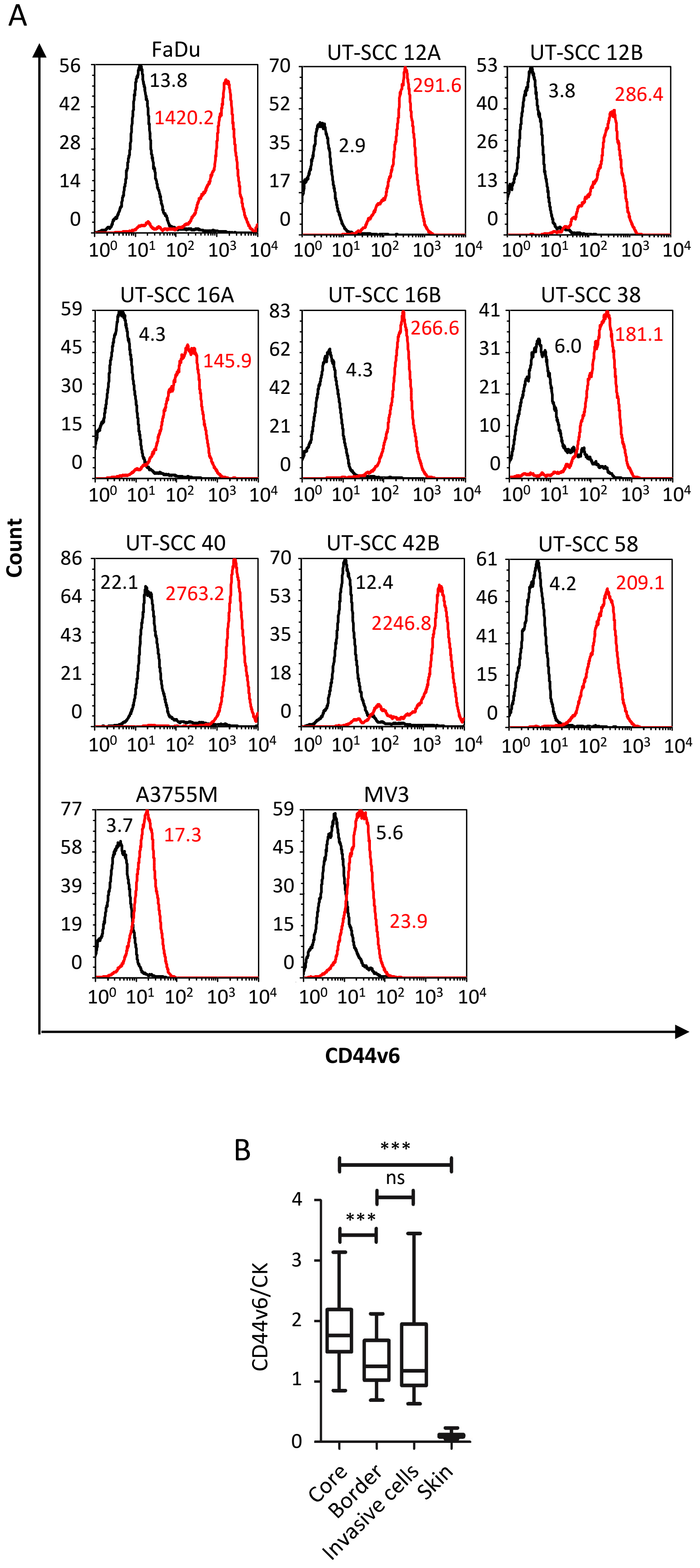

Supplementary Figure 3

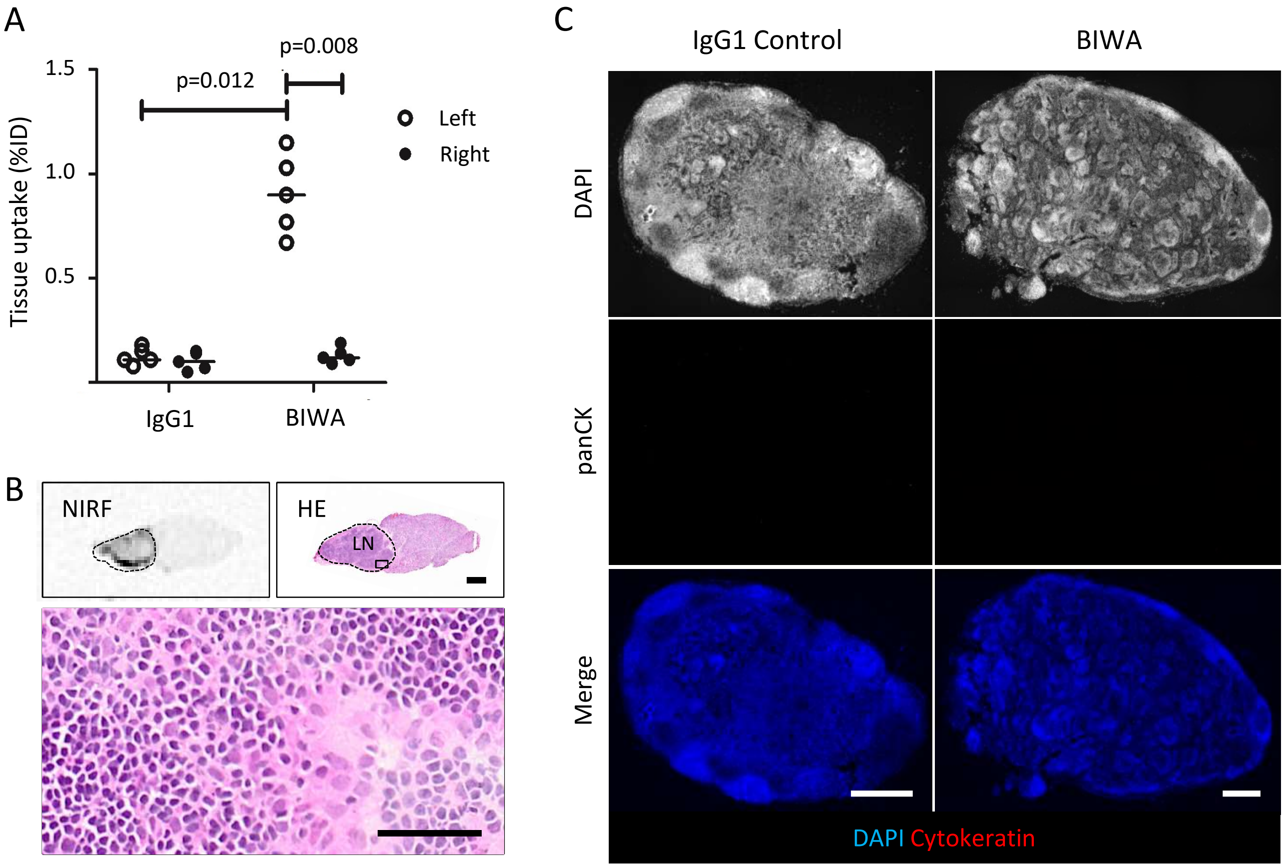

Supplementary Figure 4

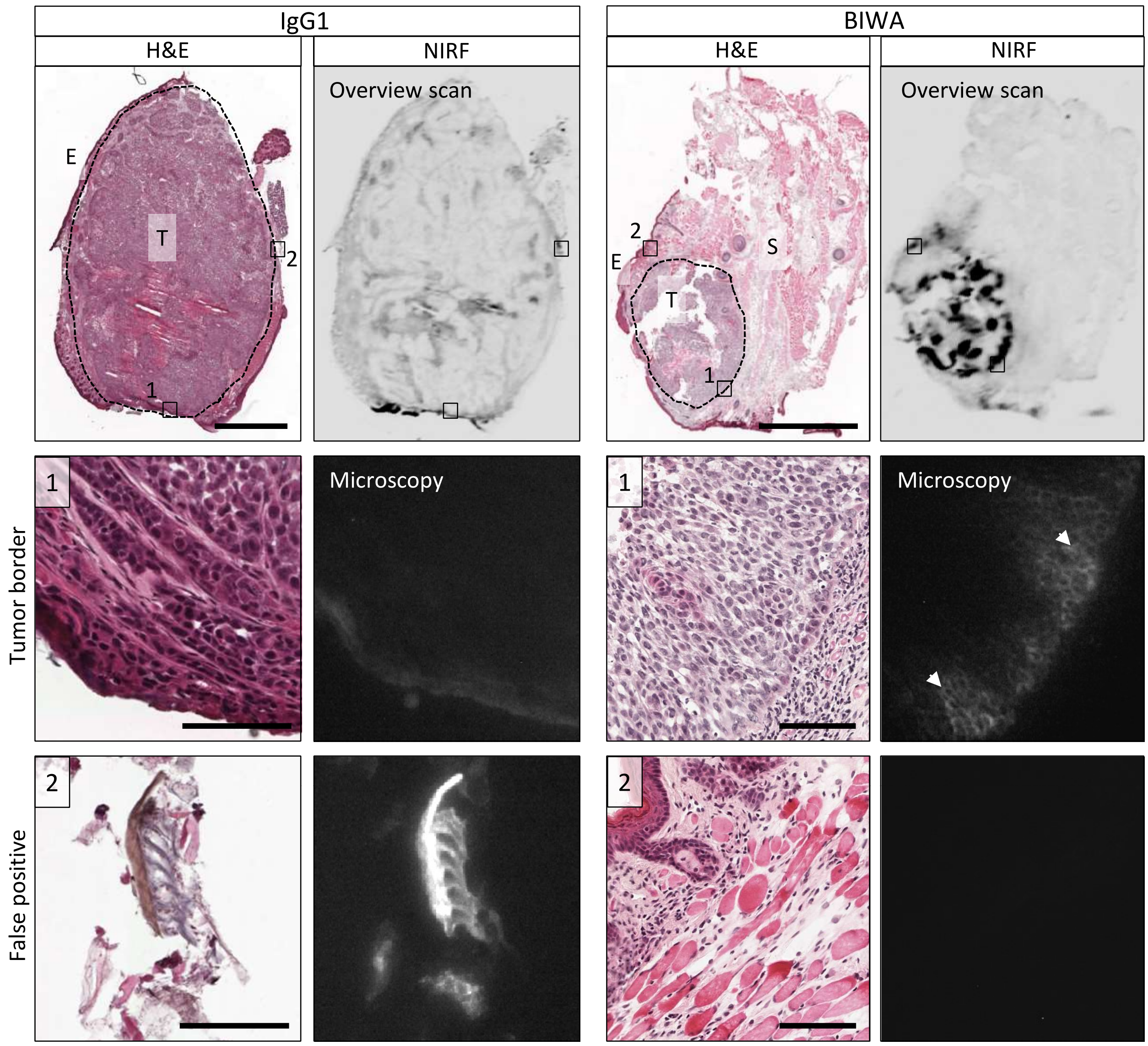

Figure legends

Supplementary figure 1. *In situ* detection of candidate targets for FGS in human HNSCC tumors. **A** Representative primary HNSCC lesion showing expression of c-Met, EMMPRIN (weak expression), E-cadherin and EpCAM. Tumor (T), normal epithelium (E), stroma (S). Scale bars indicate 1000 µm (overview) and 100 µm (zoom). **B** Scoring of expression level in 4-7 primary HNSCC lesions based on immunohistochemical staining.

Supplementary figure 2. Expression of CD44v6 in HNSCC and other cell lines cultured *in vitro* and UT-SCC58 tumors *in vivo*. **A** Surface expression of CD44v6 in 9 HNSCC and 2 melanoma cell lines maintained in liquid culture, detected by flow cytometry for IgG1 control (black lines) and CD44v6 (red lines). Numbers indicate the geometric mean. **B** Immunofluorescence whole-region analysis of Fig. 4D. Co-localization of CD44v6 and pan-cytokeratin (CK) staining in different areas in UT-SCC58 tumors analyzed at day 28 after implantation. Data show the medians from 12-38 analyzed images per tumor from 4 independent tumors.

Supplementary figure 3. Radioactive and fluorescence detection in cervial lymph nodes. **A** Biodistribtion expressed as percentage injected dose of ^{111}In-DTPA-BIWA-IRDye800CW and ^{111}In-DTPA-IgG1-IRDye800CW in right and left cervical lymph nodes (LN). **B** Central section and NIRF signal of a left cervical LN of a mouse injected with dual-labeled BIWA. Dotted line marks the LN edge. Scale bars indicate 500 µm (overview) and 50 µm (zoom). **C** Detection of CK in left cervical LNs. Scale bars indicate 500 µm.

Supplementary figure 4. HE staining and NIRF signals in tumors injected with dual-labeled BIWA and IgG1 isotypic control. Central, serial sections of mouse cheeks (H&E staining and NIRF signal) indicating the tumor border (dotted line; T), normal epithelium (E) and stroma (S); Left zoomed images: (1) tumor border and (2) false positive NIRF signal. Right zoomed images: (1) positive signal from tumor cells and (2) negative background from tumor stroma. Arrowheads indicate membranous staining. Scale bars indicate 2 mm (overview) and 100 µm (zoom).

Supplementary table 1. Literature survey on proteins expressed in HNSCC and application for medical or intraoperative imaging.

Marker	Prevalence (%) in HNSCC	Expression level and distribution in tumor cells	Pattern in non-tumor tissue	Imaging application	Used antibody#	References
**C-Met / Hepatocyte Growth Factor Receptor (HGFR)**	~ 62 %	Upregulated in primary tumor; upregulated in invasive front; pos. in metastasis; strong cytoplasmic signal	Weak expression mainly in basal layers of epithelium; mainly neg. in stroma	-	-	[1-9]
**Carcinoembryonic antigen (CEA)**	~ 69 %	Heterogeneous; stronger staining in differentiated and keratinized cells	Neg	-	-	[1, 10-13]
**CD44**	~ 77 %	Slightly diminished in poorly differentiated carcinomas; decreased in tongue carcinoma; predominantly found at invasive front of tumor; higher in metastasis	Expression in normal epithelium and lymphocytes	-	-	[14-17]
**CD44v6**	~ 97 %	Pos in metastasis; no sig. difference to invasive front; slightly increased, but diminished in poorly differentiated carcinomas	Expression mainly in basal layers of epithelium and endothelium	Preclinical [18-23]<br>Clinical [24-26]	U36/Bivatuzumab	[17, 27-32]
**E-cadherin**	~ 74 %	Higher expression at tumor centre;<br>neg in poor differentiated tumors	Strong expression in normal epithelium	-	-	[33-41]
**Epidermal Growth Factor Receptor (EGFR)**	~ 85 %	Pos in paired metastasis;<br>stronger at invasive front	Expression mainly in basal layers of epithelium	Preclinical [42-49]*<br>Clinical [50]*	Cetuximab, Panitumumab	[51-60]
**Epithelial Cell Adhesion Molecule (EpCAM)**	~ 61 %	Heterogeneous; diminished in metastasis	Neg in normal squamous epithelium; pos in hyperplasia	-	-	[61-64]
**Extracellular Matrix Metalloproteinase Inducer (EMMPRIN) / CD147 / Basigin**	~ 76 %	Higher in metastatic tumors; partially higher at invasive front	Low in mucosa	Preclinical [65, 66]	-	[67-69]
**Insulin-like growth factor receptor (IGFR)**	~ 58 %	-	-	-	-	[70-73]
**Mucin-1**	~ 74 %	Heterogeneous; pos in metastasis; strong cytoplasmic staining	Low expression in mucosa	-	-	[74-77]
**Vascular Endothelial Growth Factor (VEGF)(-A)**	~ 78 %	Heterogeneous	Expressed in epithelium, endothelium and immune cells	Preclinical [78]	Bevacizumab	[79-83]

* Only fluorescence imaging included

# For epitope-based preclinical or clinical targeting, used antibodies are mentioned.

References

1. Young, M.R., et al., *Oral premalignant lesions induce immune reactivity to both premalignant oral lesions and head and neck squamous cell carcinoma.* Cancer Immunol Immunother, 2007. **56**(7): p. 1077-86.
2. Yucel, O.T., A. Sungur, and S. Kaya, *c-met overexpression in supraglottic laryngeal squamous cell carcinoma and its relation to lymph node metastases.* Otolaryngol Head Neck Surg, 2004. **130**(6): p. 698-703.
3. Lim, Y.C., et al., *Overexpression of c-Met promotes invasion and metastasis of small oral tongue carcinoma.* Oral Oncol, 2012. **48**(11): p. 1114-9.
4. Cortesina, G., et al., *Staging of head and neck squamous cell carcinoma using the MET oncogene product as marker of tumor cells in lymph node metastases.* Int J Cancer, 2000. **89**(3): p. 286-92.
5. Kim, C.H., et al., *c-Met expression as an indicator of survival outcome in patients with oral tongue carcinoma.* Head Neck, 2010. **32**(12): p. 1655-64.
6. Chau, N.G., et al., *The association between EGFR variant III, HPV, p16, c-MET, EGFR gene copy number and response to EGFR inhibitors in patients with recurrent or metastatic squamous cell carcinoma of the head and neck.* Head Neck Oncol, 2011. **3**: p. 11.
7. Galeazzi, E., et al., *Detection of MET oncogene/hepatocyte growth factor receptor in lymph node metastases from head and neck squamous cell carcinomas.* Eur Arch Otorhinolaryngol, 1997. **254 Suppl 1**: p. S138-43.
8. Knowles, L.M., et al., *HGF and c-Met participate in paracrine tumorigenic pathways in head and neck squamous cell cancer.* Clin Cancer Res, 2009. **15**(11): p. 3740-50.
9. Seiwert, T.Y., et al., *The MET receptor tyrosine kinase is a potential novel therapeutic target for head and neck squamous cell carcinoma.* Cancer Res, 2009. **69**(7): p. 3021-31.
10. Yanagawa, T., et al., *Immunohistochemical demonstration of carcinoembryonic antigen (CEA) on tissue sections from squamous cell head and neck cancer and plasma CEA levels of the patients.* Int J Oral Maxillofac Surg, 1986. **15**(3): p. 296-306.
11. Kass, E.S., et al., *Carcinoembryonic antigen as a target for specific antitumor immunotherapy of head and neck cancer.* Cancer Res, 2002. **62**(17): p. 5049-57.
12. Banks, E.R., et al., *Basaloid squamous cell carcinoma of the head and neck. A clinicopathologic and immunohistochemical study of 40 cases.* Am J Surg Pathol, 1992. **16**(10): p. 939-46.
13. Argenzio, V., et al., *Radioimmunoguided surgery in squamous cell carcinoma.* Plast Reconstr Surg, 1999. **103**(2): p. 749-51.
14. Chen, J., et al., *Significance of CD44 expression in head and neck cancer: a systemic review and meta-analysis.* BMC Cancer, 2014. **14**: p. 15.
15. Krump, M. and J. Ehrmann, *Differences in CD44s expression in HNSCC tumours of different areas within the oral cavity.* Biomed Pap Med Fac Univ Palacky Olomouc Czech Repub, 2013. **157**(4): p. 280-3.
16. Sterz, C.M., et al., *A basal-cell-like compartment in head and neck squamous cell carcinomas represents the invasive front of the tumor and is expressing MMP-9.* Oral Oncol, 2010. **46**(2): p. 116-22.
17. Mack, B. and O. Gires, *CD44s and CD44v6 expression in head and neck epithelia.* PLoS One, 2008. **3**(10): p. e3360.
18. Haylock, A.K., et al., *In vivo characterization of the novel CD44v6-targeting Fab fragment AbD15179 for molecular imaging of squamous cell carcinoma: a dual-isotope study.* EJNMMI Res, 2014. **4**(1): p. 11.
19. Sandstrom, K., et al., *A novel CD44v6 targeting antibody fragment with improved tumor-to-blood ratio.* Int J Oncol, 2012. **40**(5): p. 1525-32.
20. Sandstrom, K., et al., *Targeting CD44v6 expressed in head and neck squamous cell carcinoma: preclinical characterization of an 111In-labeled monoclonal antibody.* Tumour Biol, 2008. **29**(3): p. 137-44.

21. Cheng, J., et al., *The use of closo-dodecaborate-containing linker improves targeting of HNSCC xenografts with radioiodinated chimeric monoclonal antibody U36.* Mol Med Rep, 2010. **3**(1): p. 155-60.
22. Verel, I., et al., *Long-lived positron emitters zirconium-89 and iodine-124 for scouting of therapeutic radioimmunoconjugates with PET.* Cancer Biother Radiopharm, 2003. **18**(4): p. 655-61.
23. Vermeulen, J.F., et al., *Near-infrared fluorescence molecular imaging of ductal carcinoma in situ with CD44v6-specific antibodies in mice: a preclinical study.* Mol Imaging Biol, 2013. **15**(3): p. 290-8.
24. Postema, E.J., et al., *Dosimetric analysis of radioimmunotherapy with 186Re-labeled bivatuzumab in patients with head and neck cancer.* J Nucl Med, 2003. **44**(10): p. 1690-9.
25. Stroomer, J.W., et al., *Safety and biodistribution of 99mTechnetium-labeled anti-CD44v6 monoclonal antibody BIWA 1 in head and neck cancer patients.* Clin Cancer Res, 2000. **6**(8): p. 3046-55.
26. Borjesson, P.K., et al., *Performance of immuno-positron emission tomography with zirconium-89-labeled chimeric monoclonal antibody U36 in the detection of lymph node metastases in head and neck cancer patients.* Clin Cancer Res, 2006. **12**(7 Pt 1): p. 2133-40.
27. Nestor, M., et al., *Quantification of CD44v6 and EGFR expression in head and neck squamous cell carcinomas using a single-dose radioimmunoassay.* Tumour Biol, 2007. **28**(5): p. 253-63.
28. Rodrigo, J.P., et al., *Expression of E-cadherin, CD44s, and CD44v6 in laryngeal and pharyngeal carcinomas.* Am J Otolaryngol, 2003. **24**(6): p. 384-9.
29. van Hal, N.L., et al., *Characterization of CD44v6 isoforms in head-and-neck squamous-cell carcinoma.* Int J Cancer, 1999. **82**(6): p. 837-45.
30. Herold-Mende, C., et al., *Expression of CD44 splice variants in squamous epithelia and squamous cell carcinomas of the head and neck.* J Pathol, 1996. **179**(1): p. 66-73.
31. Gotoda, T., et al., *Expression of CD44 variants and prognosis in oesophageal squamous cell carcinoma.* Gut, 2000. **46**(1): p. 14-9.
32. Fonseca, I., et al., *Expression of CD44 isoforms in squamous cell carcinoma of the border of the tongue: A correlation with histological grade, pattern of stromal invasion, and cell differentiation.* J Surg Oncol, 2001. **76**(2): p. 115-20.
33. Kim, K.H., et al., *The clinicopathological significance of epithelial mesenchymal transition associated protein expression in head and neck squamous cell carcinoma.* Korean J Pathol, 2014. **48**(4): p. 263-9.
34. Muller, S., et al., *Distinctive E-cadherin and epidermal growth factor receptor expression in metastatic and nonmetastatic head and neck squamous cell carcinoma: predictive and prognostic correlation.* Cancer, 2008. **113**(1): p. 97-107.
35. Eriksen, J.G., et al., *Expression of integrins and E-cadherin in squamous cell carcinomas of the head and neck.* APMIS, 2004. **112**(9): p. 560-8.
36. Li, J.J., et al., *Reduced E-cadherin expression is associated with lymph node metastases in laryngeal squamous cell carcinoma.* Auris Nasus Larynx, 2012. **39**(2): p. 186-92.
37. Katada, K., et al., *Plectin promotes migration and invasion of cancer cells and is a novel prognostic marker for head and neck squamous cell carcinoma.* J Proteomics, 2012. **75**(6): p. 1803-15.
38. Sakamoto, K., et al., *Overexpression of SIP1 and downregulation of E-cadherin predict delayed neck metastasis in stage I/II oral tongue squamous cell carcinoma after partial glossectomy.* Ann Surg Oncol, 2012. **19**(2): p. 612-9.
39. Nijkamp, M.M., et al., *Expression of E-cadherin and vimentin correlates with metastasis formation in head and neck squamous cell carcinoma patients.* Radiother Oncol, 2011. **99**(3): p. 344-8.
40. Huber, G.F., et al., *Down regulation of E-Cadherin (ECAD) - a predictor for occult metastatic disease in sentinel node biopsy of early squamous cell carcinomas of the oral cavity and oropharynx.* BMC Cancer, 2011. **11**: p. 217:1-8.

41. Massarelli, E., et al., *Loss of E-cadherin and p27 expression is associated with head and neck squamous tumorigenesis.* Cancer, 2005. **103**(5): p. 952-9.
42. Yang, K., et al., *In-vivo imaging of oral squamous cell carcinoma by EGFR monoclonal antibody conjugated near-infrared quantum dots in mice.* Int J Nanomedicine, 2011. **6**: p. 1739-45.
43. Helman, E.E., et al., *Optical imaging predicts tumor response to anti-EGFR therapy.* Cancer Biol Ther, 2010. **10**(2): p. 166-71.
44. van Driel, P.B., et al., *Intraoperative fluorescence delineation of head and neck cancer with a fluorescent anti-epidermal growth factor receptor nanobody.* Int J Cancer, 2014. **134**(11): p. 2663-73.
45. Keereweer, S., et al., *Optical imaging of oral squamous cell carcinoma and cervical lymph node metastasis.* Head Neck, 2012. **34**(7): p. 1002-8.
46. Day, K.E., et al., *Preclinical comparison of near-infrared-labeled cetuximab and panitumumab for optical imaging of head and neck squamous cell carcinoma.* Mol Imaging Biol, 2013. **15**(6): p. 722-9.
47. Heath, C.H., et al., *Use of panitumumab-IRDye800 to image cutaneous head and neck cancer in mice.* Otolaryngol Head Neck Surg, 2013. **148**(6): p. 982-90.
48. Heath, C.H., et al., *Use of panitumumab-IRDye800 to image microscopic head and neck cancer in an orthotopic surgical model.* Ann Surg Oncol, 2012. **19**(12): p. 3879-87.
49. Terwisscha van Scheltinga, A.G., et al., *Intraoperative near-infrared fluorescence tumor imaging with vascular endothelial growth factor and human epidermal growth factor receptor 2 targeting antibodies.* J Nucl Med, 2011. **52**(11): p. 1778-85.
50. de Boer, E., et al., *In Vivo Fluorescence Immunohistochemistry: Localization of Fluorescently Labeled Cetuximab in Squamous Cell Carcinomas.* Sci Rep, 2015. **5**: p. 10169.
51. Sheikh Ali, M.A., et al., *Expression and mutation analysis of epidermal growth factor receptor in head and neck squamous cell carcinoma.* Cancer Sci, 2008. **99**(8): p. 1589-94.
52. Xia, W., et al., *Combination of EGFR, HER-2/neu, and HER-3 is a stronger predictor for the outcome of oral squamous cell carcinoma than any individual family members.* Clin Cancer Res, 1999. **5**(12): p. 4164-74.
53. Silva, S.D., et al., *ErbB receptors and fatty acid synthase expression in aggressive head and neck squamous cell carcinomas.* Oral Dis, 2010. **16**(8): p. 774-80.
54. Wei, Q., et al., *EGFR, HER2, and HER3 expression in laryngeal primary tumors and corresponding metastases.* Ann Surg Oncol, 2008. **15**(4): p. 1193-201.
55. Sarkis, S.A., et al., *Immunohistochemical expression of epidermal growth factor receptor (EGFR) in oral squamous cell carcinoma in relation to proliferation, apoptosis, angiogenesis and lymphangiogenesis.* Head Neck Oncol, 2010. **2**: p. 13.
56. Sweeny, L., et al., *EGFR expression in advanced head and neck cutaneous squamous cell carcinoma.* Head Neck, 2012. **34**(5): p. 681-6.
57. Thariat, J., et al., *Epidermal growth factor receptor protein detection in head and neck cancer patients: a many-faceted picture.* Clin Cancer Res, 2012. **18**(5): p. 1313-22.
58. Fujii, S., et al., *Clinical significance of KRAS gene mutation and epidermal growth factor receptor expression in Japanese patients with squamous cell carcinoma of the larynx, oropharynx and hypopharynx.* Int J Clin Oncol, 2013. **18**(3): p. 454-63.
59. Rossle, M., et al., *EGFR expression and copy number changes in low T-stage oral squamous cell carcinomas.* Histopathology, 2013. **63**(2): p. 271-8.
60. Grobe, A., et al., *Immunohistochemical and FISH analysis of EGFR and its prognostic value in patients with oral squamous cell carcinoma.* J Oral Pathol Med, 2014. **43**(3): p. 205-10.
61. Murakami, N., et al., *Expression of EpCAM and prognosis in early-stage glottic cancer treated by radiotherapy.* Laryngoscope, 2014. **124**(11): p. E431-6.
62. Andratschke, M., et al., *Limited suitability of EpCAM for molecular staging of tumor borders in head and neck cancer.* Anticancer Res, 2006. **26**(1A): p. 153-8.
63. Takes, R.P., et al., *Expression of genetic markers in lymph node metastases compared with their primary tumours in head and neck cancer.* J Pathol, 2001. **194**(3): p. 298-302.

64. Yanamoto, S., et al., *Clinicopathologic significance of EpCAM expression in squamous cell carcinoma of the tongue and its possibility as a potential target for tongue cancer gene therapy.* Oral Oncol, 2007. **43**(9): p. 869-77.
65. Knowles, J.A., et al., *Molecular targeting of ultrasonographic contrast agent for detection of head and neck squamous cell carcinoma.* Arch Otolaryngol Head Neck Surg, 2012. **138**(7): p. 662-8.
66. Newman, J.R., et al., *Stereomicroscopic fluorescence imaging of head and neck cancer xenografts targeting CD147.* Cancer Biol Ther, 2008. **7**(7): p. 1063-70.
67. Rosenthal, E.L., et al., *Expression of extracellular matrix metalloprotease inducer in laryngeal squamous cell carcinoma.* Laryngoscope, 2003. **113**(8): p. 1406-10.
68. Huang, T., et al., *Correlation between expression of extracellular matrix metalloproteinase inducer and matrix metalloproteinase-2 and cervical lymph node metastasis of nasopharyngeal carcinoma.* Ann Otol Rhinol Laryngol, 2013. **122**(3): p. 210-5.
69. Andrade, A.L., et al., *Immunoexpression of EGFR and EMMPRIN in a series of cases of head and neck squamous cell carcinoma.* Pathol Res Pract, 2015.
70. Sun, J.M., et al., *Insulin-like growth factor binding protein-3, in association with IGF-1 receptor, can predict prognosis in squamous cell carcinoma of the head and neck.* Oral Oncol, 2011. **47**(8): p. 714-9.
71. Ouban, A., et al., *Expression and distribution of insulin-like growth factor-1 receptor in human carcinomas.* Hum Pathol, 2003. **34**(8): p. 803-8.
72. Dale, O.T., et al., *IGF-1R expression is associated with HPV-negative status and adverse survival in head and neck squamous cell cancer.* Carcinogenesis, 2015. **36**(6): p. 648-55.
73. Matsumoto, F., et al., *Relationship between insulin-like growth factor-1 receptor and human papillomavirus in patients with oropharyngeal cancer.* Head Neck, 2015. **37**(7): p. 977-81.
74. Croce, M.V., et al., *MUC1 mucin and carbohydrate associated antigens as tumor markers in head and neck squamous cell carcinoma.* Pathol Oncol Res, 2001. **7**(4): p. 284-91.
75. Croce, M.V., et al., *Differential expression of MUC1 and carbohydrate antigens in primary and secondary head and neck squamous cell carcinoma.* Head Neck, 2008. **30**(5): p. 647-57.
76. Rabassa, M.E., et al., *MUC1 expression and anti-MUC1 serum immune response in head and neck squamous cell carcinoma (HNSCC): a multivariate analysis.* BMC Cancer, 2006. **6**: p. 253.
77. Jeannon, J.P., et al., *Expression of MUC1 and MUC2 glycoproteins in laryngeal cancer.* Clin Otolaryngol Allied Sci, 2001. **26**(2): p. 109-12.
78. Withrow, K.P., et al., *Assessment of bevacizumab conjugated to Cy5.5 for detection of head and neck cancer xenografts.* Technol Cancer Res Treat, 2008. **7**(1): p. 61-6.
79. Tse, G.M., et al., *Strong immunohistochemical expression of vascular endothelial growth factor predicts overall survival in head and neck squamous cell carcinoma.* Ann Surg Oncol, 2007. **14**(12): p. 3558-65.
80. Kyzas, P.A., et al., *Prognostic significance of VEGF immunohistochemical expression and tumor angiogenesis in head and neck squamous cell carcinoma.* J Cancer Res Clin Oncol, 2005. **131**(9): p. 624-30.
81. Ninck, S., et al., *Expression profiles of angiogenic growth factors in squamous cell carcinomas of the head and neck.* Int J Cancer, 2003. **106**(1): p. 34-44.
82. Bowden, J., et al., *Expression of vascular endothelial growth factor in basal cell carcinoma and cutaneous squamous cell carcinoma of the head and neck.* J Cutan Pathol, 2002. **29**(10): p. 585-9.
83. Henriques, A.C., et al., *Immunohistochemical expression of MMP-9 and VEGF in squamous cell carcinoma of the tongue.* J Oral Sci, 2012. **54**(1): p. 105-11.
